# Non-Conventional Yeast: Behavior under Pure Culture, Sequential and Aeration Conditions in Beer Fermentation

**DOI:** 10.3390/foods11223717

**Published:** 2022-11-18

**Authors:** Vanesa Postigo, Tadhg O’Sullivan, Tom Elink Schuurman, Teresa Arroyo

**Affiliations:** 1 Department of Agri-Food, Madrid Institute for Rural, Agriculture and Food Research and Development (IMIDRA), El Encín, A-2, km 38.2, 28805 Alcalá de Henares, Spain; 2 Brewery La Cibeles, Petróleo 34, 28918 Leganés, Spain; 3 Heineken Supply Chain B.V., Burgemeester Smeetsweg 1, 2382 PH Zoeterwoude, The Netherlands

**Keywords:** non-conventional yeasts, non-*Saccharomyces*, ale beer, aeration, aromas

## Abstract

The use of wild yeasts, isolated from different environments, is becoming the most interesting option for the production of new beers. The objective of this study is to evaluate the potential of seven non-conventional yeast strains from five different species (*Saccharomyces cerevisiae*, *Hanseniaspora guilliermondii*, *Metschnikowia pulcherrima*, *Torulaspora delbrueckii*, and *Zygosaccharomyces bailii*) isolated from Madrid agriculture to produce type ale beer. Wild yeast strains were evaluated at laboratory and pilot plant scales under different fermentation conditions (pure, aerated, and sequential culture). Strain *S. cerevisiae* SafAle S-04 was used as a reference. Throughout the fermentation of beer, volatile compounds were determined by GC and residual sugars by HPLC, among other parameters. The yeast strains used for the fermentation in pure culture conditions were unable to ferment maltose and maltotriose (0.73–1.18% *v*/*v* of ethanol). The results of the study under aerated conditions showed varying levels of higher alcohol and ester concentrations. It should be noted that the strain CLI 1057 (*S. cerevisiae*) fermented maltose in the presence of oxygen (Kluyver effect). This strain also showed a high production of 4-vinyl guaiacol, making it suitable for producing beers with a phenolic profile. Finally, three strains (*H. guilliermondii*, *Z. bailii,* and *T. delbrueckii*) were evaluated in sequential culture together with commercial strain and found to improve the organoleptic characteristics of the brewed beer. These approaches offer the opportunity to add new product characteristics to the beers.

## 1. Introduction

Beer drinking habits have changed over the last few years. The volume of beer produced grew enormously until 2013, when it started to decline, with the production in 2021 amounting to about 1.86 billion hL [1]. In this context, the craft beer industry has taken an important role in the market. Among other proposals, a commitment to the production of non-alcoholic and low-alcoholic beers is emerging [2]. Therefore, there is general importance in developing new products that meet the needs of consumers both in terms of flavors and healthier products. In this sense, interest has focused on non-conventional yeasts and the search for species and strains with low ethanol production due to their low fermentative capacity and new aromatic profiles [2,3,4].

Low ethanol production is often determined by the inability of some yeasts to ferment maltose, the major sugar in beer wort. Steensels and Verstrepen conducted a study with wild *Saccharomyces* yeast strains, where only 12% could ferment 50% of the available sugars in the wort [5]. On the other hand, the work by Methner et al., with 110 non-*Saccharomyces* yeasts showed that only 30% of the yeasts studied were able to ferment 25% of the maltose and maltotriose content of the wort [6]. It should be noted that not only is species screening important, but so is strain screening, as the ability to ferment the sugars in wort is often strain-dependent.

Species such as *Torulaspora delbrueckii*, *Wickerhamomyces anomalus*, *Hanseniaspora guilliermondii*, *Schizosaccharomyces pombe*, *Metschnikowia pulcherrima*, *Brettanomyces* spp., *Lachancea* spp., have been widely described and used in beer fermentation, not only for the organoleptic characteristics that many of them provide, but also for their potential for the production of low ethanol beers due to their inability to ferment maltose and maltotriose [7,8,9,10,11].

The limitation of some yeasts to ferment sugars makes it possible to use certain species and strains sequentially with *Saccharomyces cerevisiae*, improving the flavor and reducing the sweetness of beers due to residual sugars. In this regard, the studies on wine are considerably more abundant than on beer. However, work carried out by Holt and Bourbon-Melo et al., has shown beers obtained under sequential fermentation conditions with yeasts of the genera *Torulaspora*, *Pichia*, *Brettanomyces*, *Zygotorulaspora,* and *Hanseniaspora* [12,13].

From a technological point of view, the application of aeration during fermentation is an interesting tool for controlling yeast metabolism during fermentation [14]. Firstly, because of Crabtree-negative yeasts, when the oxygen concentration in the medium is saturated, the yeast metabolism starts to be predominantly oxidative, thus reducing the ethanol content and increasing the yeasts’ biomass. On the other hand, aeration can also affect aroma compounds by reducing or increasing their concentration in beer (the content of acetaldehyde could decrease, and the concentration of higher alcohols increase) [15,16,17].

In this context, the isolation, selection, and aroma characterization of non-conventional yeasts are key tools for the development of novel beers. This research aimed to evaluate the fermentation capacity of seven non-conventional yeast (one *Saccharomyces* and six non-*Saccharomyces* strains) under different conditions: pure culture, fermentation with aeration, and sequential fermentation, to enhance beer flavor and to obtain low alcohol beers.

## 2. Materials and Methods

### 2.1. Yeast Strains and Wort

The study was performed at Heineken Research and Development Department using the Madrid Institute for Rural, Agriculture and Food Research and Development (IMIDRA, Madrid, Spain) collection of native wine yeast from D.O. “Vinos de Madrid”. These strains were isolated from Madrid’s wine, grapes, must, vineyard and cellars and then cryogenically preserved at −80 °C (YPD broth supplemented with 40% (*w*/*v*) glycerol). One *Saccharomyces cerevisiae* strain (CLI 1057) and six non-*Saccharomyces* yeast strains were studied (CLI 101, *Metschnikowia pulcherrima*, CLI 512, *Hanseniaspora guilliermondii*, CLI 622 and CLI 691, *Zygosaccharomyces bailii*, CLI 895 and CLI 901, *Torulaspora delbrueckii*). The yeast strains tested in this study were previously selected from 141 *Saccharomyces* and 77 non-*Saccharomyces* yeast strains, mainly for having a low fermentative capacity and producing outstanding aromas [18,19]. These trials were conducted at IMIDRA Research Centre (Madrid, Spain). The *Saccharomyces cerevisiae* commercial strain SafAle S-04 (Fermentis, Lesaffre, France) was used as the control during the fermentation trials.

To ensure the identity and purity of all the strains, *Saccharomyces* yeasts were tested by microsatellite multiplex PCR using the highly polymorphic loci SC8132X, YOR267C, and SCPTSY7 [20]. At the same time, non-*Saccharomyces* were examined by amplifying the 5.8S rRNA gene and the two ribosomal internal transcribed spacers, using the primer pair ITS1/ITS4 [21], followed by restriction with Hae III, Cfo I, and Hinf I [22].

To perform the different fermentations, a 100% malt-hopped wort brewed at Heineken was used (16.6 °P).

### 2.2. Lab-Scale Screening

Fermentations were carried out in 1 L flasks containing 400 mL of wort under rotatory shaking at 100 rpm and 20 °C, with daily weight loss monitoring until stabilization. Yeast pre-cultures were grown in YPD broth on a rotary shaker (100 rpm) for 24 h at 28 °C. Suspensions of 10^6^ cells mL^−1^ of pre-cultures of each strain, including the commercial strain, were used to inoculate the wort.

Daily samples were taken to analyze fermentable sugars and esters, and a final sample was used to measure the final ethanol production. Strains that produced off-flavors for beer (by direct olfaction or high concentrations obtained in the analyses) and those that did not ferment were discarded (CLI 901, CLI 691). All the fermentations were carried out in duplicate and used as a reference for the commercial yeast strain S-04.

### 2.3. Pilot Plant Scale: 7 L

Based on the results obtained from the lab-scale screening, four strains (CLI 101, CLI 622, CLI 895, and CLI 1057) were selected and tested on a larger scale (7 L) under the conditions established for the industrial study. The CLI 512 strain (*Hanseniaspora guilliermondii*), already tested in previous studies [19], was also included in order to contrast the results in different species. Batch cultivations under industrial conditions (experiments A, B, and C) were performed in 10 L stirred stainless-steel fermenters containing 7 L of 16.6 °Plato wort and a temperature of 18 °C. For experiment A, the fermentation was performed by pure cultures under anaerobic conditions with the yeast strains CLI 101, CLI 512, CLI 622, CLI 895, and CLI 1057 versus the commercial strain S-04. In experiment B, carried out with strains CLI 101, CLI 512, CLI 622, CLI 895, and CLI 1057, the fermentation was aerated continuously during the first 48 h with sterile O_2_ (1.5 L O_2_ L^−1^) once the inoculum was added. After aeration, fermentation was performed under anaerobic conditions until stabilization. The purpose of this experiment was to determine if any of the strains exhibited negative Crabtree behavior, in order to reduce the ethanol content of the beers. In experiment C, the aim was to enhance the flavor of the beers by means of sequential fermentation. The non-*Saccharomyces* strains (CLI 512, CLI 622, CLI 895) were inoculated and fermented for the first four days, and then the commercial yeast *Saccharomyces* S-04 was added. Pre-cultures were incubated on a shaker (100 rpm, YPD broth) for 24 h at 28 °C, and the total pitching rate was 2.8–5.9 x 10^6^ cells mL^−1^.

### 2.4. Beer Analysis

Samples were taken daily from both laboratory (400 mL) and pilot plant scale (7 L) fermentations for analysis of apparent extract, ethanol, sugars, esters, higher alcohols, acetaldehyde, and dimethyl sulfide (DMS). In the 7 L fermentations, vicinal diketones (diacetyl and 2,3-pentanedione), 4-vinyl guaiacol (4VG), and yeast viability were also determined. All analyses were carried out by the Quality Assurance Laboratories (QAL, Heineken, Zoeterwoude, The Netherlands).

The ethanol and extract content of beer samples were measured with an Anton Paar Alcolyzer Plus beer analyzer (Graz, Austria). Fermentable sugars were analyzed by Acquity UPLC-RI system (Waters) using Acquity UPLC BEH Amide 1.7 μm; 2.1 × 150 mm column and Acquity UPLC BEH Amide VanGuard 1.7 μm; 2.1 × 5 mm guard column (Waters). The oven temperature was 65 °C, the flow cell temperature was 40 °C, and the mobile phase was acetonitrile/bidistilled water (75:25), with a flow rate of 0.2 mL min^−1^. The chromatographic analysis was performed according to EBC method 8.7 [23]. All samples were filtered prior to analyses with Millex Samplicity Filters; 0.20 μm Hydrophilic PTFE (Millipore Corp., Bedford, MA, USA).

Volatile compounds (esters, higher alcohols, acetaldehyde, and DMS) were determined according to EBC method 9.39 [24] using gas chromatograph Trace 1300 (GC-FID, Interscience, Breda, The Netherlands) equipped with a flame ionization detector and DBWaxETR, 0.32 mm ID × 60 m column. Concentrations were determined by calibration lines for each compound.

Vicinal diketones were analyzed according to EBC method 9.24.2 by GC-ECD [24], with Trace 1300 gas chromatograph (Interscience, Breda, The Netherlands) and CP-Sil 8 CB (0.32 mm ID × 50 m) fused silica WCOT column.

Determination of 4VG was performed by high-performance liquid chromatography (HPLC) using the Acquity UPLC system (Waters) equipped with a fluorescence detector, Acquity UPLC BEH C18 (17 μm; 2.1 × 150 mm) column (Waters) and Acquity UPLC BEH C18 (1.7 μm; 2.1 × 5 mm) guard column.

To determine the viability and number of the yeast cells during the whole fermentation process, the Yeast Cell NucleoCounter YC-100 (ChemoMetec A/S, Allerød, Denmark) was used.

### 2.5. Statistical Analysis

Significant differences in the data were determined with one-way analysis of variance (ANOVA) and Tukey post-hoc test using R Studio 4.1 (Integrated Development for R. RStudio, PBC, Boston, MA, USA) at the 95% confidence level. All reported values were obtained from the analysis of duplicate fermentations and are represented as mean ± standard deviation.

## 3. Results and Discussion

### 3.1. Preliminary Screening

The evaluation of the six non-conventional yeast strains (CLI 101, CLI 622, CLI 691, CLI 901, CLI 895, and CLI 1057) was conducted to determine their fermentation performances for beer production. Table 1 shows the data for the main analytical characteristics of the resulting beers. All yeasts, with the exception of the *Saccharomyces cerevisiae* control strain S-04 and *Torulaspora delbrueckii* strain CLI 901, fermented the wort to levels of 1.07–1.26% (*v*/*v*) of ethanol after four days, with an apparent attenuation of 16.18–22.06%. As expected, the best fermentation performance corresponded to *S. cerevisiae* commercial strain S-04 since its ethanol production was 5.69% (*v*/*v*) and 88.24% of attenuation. The strain CLI 901 was unable to ferment the wort and therefore discarded for the next larger-scale trial.

One of the main and undoubted differences between yeasts is their ability to ferment the different sugars present in the wort. The study strains fermented the monosaccharides (glucose and fructose) and/or sucrose present in the wort while demonstrating no significant consumption of either maltose or maltotriose. The inability of this species to metabolize maltose, the principal sugar present in the wort, makes them especially attractive for the production of low-alcohol beers. Significant differences were also found in terms of sucrose metabolism, where only strains CLI 895 (*Torulaspora delbrueckii*) and CLI 1057 (*S. cerevisiae*) fermented this sugar. On the other hand, strain CLI 101 (*Metschnikowia pulcherrima*), described as unable to ferment sucrose [25], confirmed this statement. At the same time, the species *Zygosaccharomyces bailii* can show variable sucrose fermentation [26,27], being negative for the strain used in our study.

In addition, in this preliminary screening, the total production of volatile compounds by the study strains was also evaluated (Table 2). The production of volatile higher alcohols and esters differed according to the yeast species and strain, regardless of the sugars fermented.

Levels of some volatile components such as acetaldehyde, dimethyl sulfide, acetone, diacetyl, and 2,3-pentanedione can affect the sensory properties of a beer. The concentration of these compounds depends on pitching rates, aeration levels, times of filling, fermentation temperatures, as well as the yeast strain used [28].

Production of acetaldehyde (unripe apple, pungent, fruity flavor) for *S. cerevisiae* strains (CLI 1057 and S-04) exceeded the 25 mg L^−1^ threshold with a measured value higher than 30 mg L^−1^ [29]. The strain of *T. delbrueckii* (CLI 895) also showed high values (24.45 mg L−^1^). Acetaldehyde is formed in green beer during the first three days but is reduced to ethanol during the last stages of fermentation [7,30].

DMS produced by yeasts during fermentation is formed from dimethyl sulphoxide and is more commonly found in lager beers than in ale beers [31,32,33] with a threshold value of 30 µg L^−1^. Low DMS concentrations impart positive characteristics in lager beers, but high DMS concentrations (100 µg L^−1^) result in spoilage aromas such as cooked sweetcorn [28,34]. All strains, except CLI 691, produced DMS values similar to or lower than 17 µg L^−1^. Since the beer fermented with *Z. bailii* strain CLI 691 showed values above the perception threshold and somewhat elevated for ale-type beers (>30 µg L^−1^) [34], it was discarded for further scaling.

Acetone compound presents alcohol and solvent-like aromas when the perception threshold of 100 mg L^−1^ is exceeded [35]. All studied strains, as well as the control strain, showed similar values (0.96–1.48 mg L^−1^) but well below the threshold. These values are within the average acetone values found by Postel et al., in German beer [36].

Concentrations of total higher alcohols were between 109.00 and 156.55 mg L^−1^, approximately half the concentration of the commercial strain (304.05 mg L^−1^). Values below 300 mg L^−1^ give the beer refreshing, flower, and pleasant notes, thus adding complexity to the beer [37]. However, in terms of total ester production, there are variations between species and strains, with concentrations in some cases being twice as high as in other strains (ranging between 2.17 and 7.95 mg L^−1^).

### 3.2. Pilot Plant Fermentation: 7 L

The six non-conventional yeast strains tested at laboratory scale were shown to be suitable for testing at 7 L scale in the pilot plant, with the exception of strain CLI 691 due to its high DMS production and strain CLI 901, as it was not able to ferment beer wort. Furthermore, the selected yeasts showed good levels of esters and higher alcohols, as well as pleasant aromas by direct olfaction. Strain CLI 512 of *Hanseniaspora guilliermondii* was also included in the different studies. Its selection was based on previous results obtained with this strain at IMIDRA Research Centre (low fermentative kinetics and ester production) [19] in order to be able to contrast results between different species.

**Figure 1:**
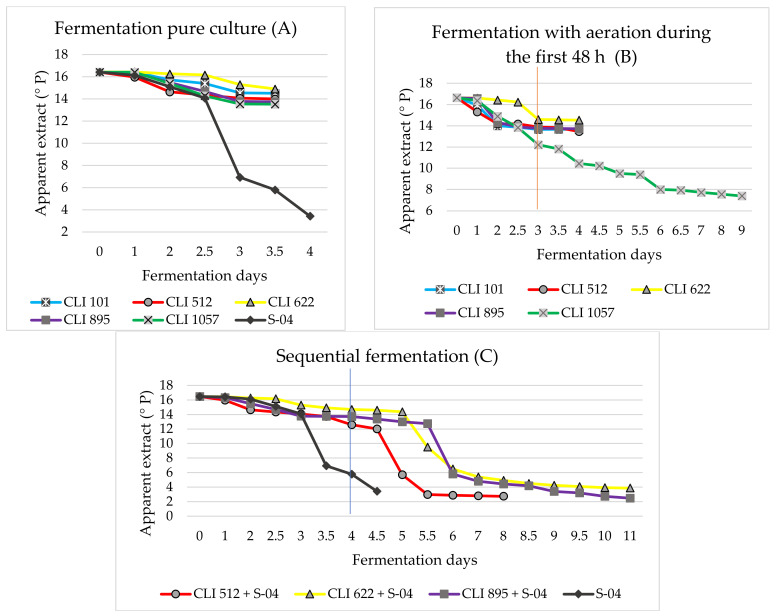
Fermentation kinetics for the different experiments: pure culture (**A**), fermentation with aeration during the first 48 h (**B**), and sequential fermentation (**C**). The vertical line in experiment B indicates the moment in which aeration was stopped, while in experiment C indicates when de *S. cerevisiae* strain S-04 was added.

Scaled-up pure culture fermentations (Experiment A) with *M. pulcherrima* (CLI 101), *H. guilliermondii* (CLI 512), *Z. bailii* (CLI 622), *T. delbrueckii* (CLI 895), *S. cerevisiae* (CLI 1057) and the reference strain *S. cerevisiae* S-04 achieved attenuation levels comparable to those produced during small-scale fermentation (Figure 1).

The highest ethanol contents were observed after 3 days of fermentation. The strains that were unable to ferment sucrose, maltose, and maltotriose (CLI 101, CLI 512, CLI 622) showed ethanol ranges of 0.73 and 0.80% (*v*/*v*). The sucrose-fermenting strains CLI 895 and CLI 1057 produced higher ethanol content ranging between 1.11 and 1.18% (*v*/*v*), respectively. S-04, the control strain, reached an ethanol content of 5.38% (*v*/*v*). After fermentation, the viability of the cells by the different strains was between 99.23 and 99.93%. Likewise, the pH reached by the non-conventional yeasts was 4.4–4.6.

In the case of experiment B, where aeration was carried out on a continuous basis (1.5 L O_2_ L^−1^) during the first 48 h of fermentation once the inoculum was added, several significant differences were observed in terms of cell concentration, as well as the fermentative capacity of one of the strains (CLI 1057). Under brewing conditions, oxygen can be considered a nutrient for yeast, as it can become a limiting factor for cell growth during propagation as well as during fermentation [38,39]. Oxygen consumption is also extremely fast during the initial lag phase of fermentation [40]. Aeration during fermentation can contribute to increased cell growth, as well as variations in beer composition or even a reduction in ethanol content [41,42]. The fermentations with aeration were somewhat more vigorous during fermentation as the sugars fermented more quickly in approximately one day. In contrast, in experiment A, they fermented in two or three days.

**Figure 2:**
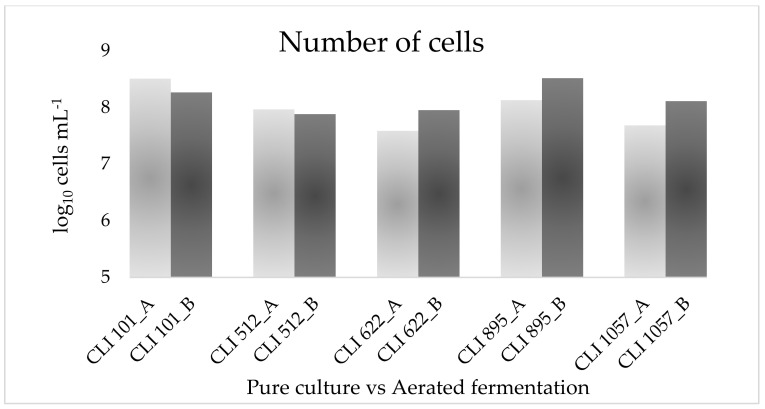
Cells concentration at the end of fermentation in pure cultures under anaerobic conditions (A, columns light grey) and fermentations with 48 h of continuous aeration (B, columns dark grey).

However, no major changes were observed in the final pH of the beers (4.5–4.9), and a small reduction in the ethanol content (0.6% (*v*/*v*) for CLI 101, CLI 512, and CLI 622, and 1% (*v*/*v*) for CLI 895), except for strain CLI 1057, whose final pH was 3.7 and ethanol produced was 3.5% (*v*/*v*). Yeast strains CLI 101 (*M. pulcherrima*) and CLI 512 (*H. guilliermondii*) showed a decrease in cells population level (1.84 × 10^8^ and 7.6 × 10^7^ cells mL^−1^, respectively) compared to the pure culture fermented anaerobically (3.22 × 10^8^ and 9.2 × 10^7^ cells mL^−1^, respectively), which could explain the lower ethanol production obtained. In contrast, CLI 622 (*Z. bailii*), CLI 895 (*T. delbrueckii*), and CLI 1057 (*S. cerevisiae*) showed a more than two-fold increase in cell growth (Figure 2). Some studies suggest that the addition of oxygen during fermentation may inhibit the growth of certain yeast strains by requiring low concentrations of oxygen for cell growth [15]. The fermentation behavior of the *S. cerevisiae* strain CLI 1057 is noteworthy since, under anaerobic conditions, it was only capable of fermenting glucose, fructose, and sucrose, whereas, under aerated conditions, this strain began to ferment part of the maltose present in wort, thus prolonging the fermentation time to 9 days as in experiment A it was only 3–4 days. This maltose assimilation could be explained because of the Kluyver effect. This effect allows some yeasts, under aerobic conditions, to assimilate sugars that they are not able to assimilate anaerobically since their transport seems to be inhibited anaerobically but not aerobically. Depending on the yeast strains, some can be Kluyver effect positive for some sugars and negative for others [43,44]. Nevertheless, although strains CLI 101, CLI 512, CLI 622, and CLI 895 showed a small decrease in ethanol content, none initially showed negative Crabtree behavior.

Regarding experiment C, sequential fermentation, the non-*Saccharomyces* strains were fermented under pure culture conditions during the first four days, at which time the *Saccharomyces* yeast S-04 was added. Combinations of non-conventional yeast species and *S. cerevisiae* mostly showed similar slow fermentation progress (10–11 days of fermentation) for strains CLI 622 and CLI 895 (*Z. bailii* and *T. delbrueckii*, respectively), in comparison with the *H. guilliermondii* strain (CLI 512) which showed faster fermentation (7–8 days) (Figure 1). The slower fermentation for strains CLI 622 and CLI 895 could be due to the inhibition of this species by *S. cerevisiae* after inoculation at 96 h of fermentation. Non-*Saccharomyces* yeasts can inhibit the growth of *S. cerevisiae* in co-inoculated or sequential cultures as they can produce toxic compounds such as medium-chain fatty acids and killer factors that can inhibit the growth of *Saccharomyces*. Also, early nutrient consumption by non-*Saccharomyces* yeasts during the first days of fermentation can limit the subsequent growth of *Saccharomyces* yeast, as well as the fermentative capacity, once inoculated sequentially [45]. The three strains used in sequential fermentation reached fermentation levels similar to those of the control strain (apparent extract 3.43 °P) or even lower, as in the case of strains CLI 512 and CLI 895 (apparent extract 2.75 and 2.49 °P, respectively).

### 3.3. Volatile Components Analyzed in 7 L

Beers are beverages rich in alcohols and esters formed during fermentation [46]. Higher alcohols are considered the most abundant aromatic compounds in beer and are generated from amino acids through the Ehrlich route or from carbohydrate metabolism [47]. Esters are formed by yeast via an enzyme-catalyzed reaction between acyl-CoA and higher alcohols. They are an important family of compounds for the production of different beverages besides beer (wine, whiskey) due to their low threshold of perception [48]. 

Table 3 shows the volatile compounds produced by the selected yeast strains in the three conditions of the study (Experiments A, B, C). The aroma profiles of aerated fermentation and sequential fermentations showed significant differences compared to single fermentation with a species-dependent relationship. From a general point of view, fermentations carried out with continuous aeration can modify the organoleptic characteristics of beers, as ester concentrations are usually reduced, higher alcohols increase, and other compounds such as diacetyl decrease in concentration [15,16].

The total higher alcohol content was noticeably higher with aeration (from 57.4 to 335.45 mg L^−1^) and in sequential fermentation (from 132.7 to 217.5 mg L^−1^) compared to pure culture fermentation (from 30.75 to 102.25 mg L^−1^). Total ester content showed higher concentrations in aerated fermentation (from 3.89 to 50.81 mg L^−1^), except for *Z. bailii* strain CLI 622, whose value was 2.54 mg L^−1^ in pure culture and was reduced to 1.44 mg L^−1^ with aeration. The sequential fermentation reached higher values of esters (from 55.31 to 102.79 mg L^−1^) than the pure culture (from 1.95 to 8.31 mg L^−1^). As an exception, strain CLI 512 showed higher values than those observed for the control strain S-04 (102.79 and 90.12 mg L^−1^, respectively). Compounds such as acetaldehyde, DMS, acetone, and vicinal diketones (VDKs: diacetyl and 2,3-pentanedione), which produce undesirable aromas in beer, showed very different behaviors depending on the type of experiment as well as the strain.

Regarding Experiment B, fermentation with aeration, in more detail and compared to pure culture, higher alcohols such as methanol showed slightly lower concentrations, except for strain CLI 101 (*M. pulcherrima*) where it was reduced by up to half, but in no case exceeded the perception threshold (10,000 mg L^−1^) [49]. Amyl alcohols (2- and 3-methyl-1-butanol) are mainly characterized by alcoholic, banana, sweet, malty, or vinous flavors. For this experiment, the *S. cerevisiae* strain CLI 1057 exhibited the highest concentrations of these compounds (201.41 mg L^−1^) due to the Kluyver effect on maltose, while the lowest was the *M. pulcherrima* strain CLI 101 and *H. guilliermondii* strain CLI 512 (31.85 and 31.51 mg L^−1^, respectively). As the detection limit is 50–70 mg L^−1^ [50], these beers will be characterized by sweetness and fruity flavors. It is also worth highlighting the production of amyl alcohols by strain CLI 1057 in pure culture since, despite not having fermented maltose, its production exceeds the threshold of perception (70.19 mg L^−1^). It is much higher than that produced by the other strains (16.53–39.40 mg L^−1^) and is around half that produced by the control strain S-04 (120.47 mg L^−1^), which does ferment maltose. The concentration of these compounds usually ranges from 31.31 to 59.46 mg L^−1^ for lager beers [51] and from 37.19 to 140.21 mg L^−1^ for ale beers fermented with *Saccharomyces cerevisiae* yeast [52]. The highest concentration values obtained in pure culture, apart from the CLI 1057 strain, are those corresponding to the *T. delbrueckii* strain CLI 895 (39.40 mg L^−1^), whose values are similar to those obtained in the study carried out by Michel et al. [9] (29.10 mg L^−1^), taking into account the initial differences in density (12 °P). *T. delbrueckii* has been described as a higher producer of amyl alcohols [53], the non-*Saccharomyces* evaluated with the highest concentration. After aeration in the fermentation, the propanol levels increased, in some cases to a value three times higher than in the pure culture, except for strain CLI 512 (*H. guilliermondii*), in which case the concentration was similar. The same was true for isobutanol alcohol, whose concentration increased significantly, except for CLI 101. For none of these compounds (propanol and isobutanol) were the thresholds exceeded (800 mg L^−1^ and 200 mg L^−1^, respectively) [49,54]. In general, the total higher alcohols increased in the aerated culture, with the increase in strains CLI 622, CLI 895, and CLI 1057, whose concentrations were two to three times higher than those found in the non-aerated culture, being quite noticeable. This is related to the increased cell growth that occurs during aeration, as it is due to the production of higher alcohols that are linked to the amino acid metabolism of the yeast [55].

In terms of esters, aeration during the first 48 h of fermentation produced an increase of esters for strains CLI 101, CLI 512, CLI 895, and CLI 1057, being quite remarkable for strain CLI 512 due to the high production of ethyl acetate (50.58 mg L^−1^) which can provide a solvent, fruity or sweetish aroma when values are above 30 mg L^−1^ [49]. This is contrary to the studies carried out by Kirsop, where the concentration of esters decreases with aeration [15]. On the other hand, studies by Jones et al., showed that the content of ethyl acetate and isoamyl acetate increases after the addition of oxygen after 12 h of fermentation [56]. It is also worth noting the behavior of strains CLI 512 and CLI 1057 for the production of isoamyl acetate, which was drastically reduced below the perception threshold (1.2 mg L^−1^) (from 0.92 to 0.15 mg L^−1^ for CLI 512 and from 2.46 to 0.92 mg L^−1^ for CLI 1057) [49]. The decrease in isoamyl acetate concentration with aeration has also been observed in previous studies with *S. cerevisiae* yeasts carried out by Mauricio et al. [57]. For the rest of the esters analyzed (ethyl formate, ethyl propionate, and ethyl caproate), as with the higher alcohols, there were different variations in the concentrations of the compounds depending on the study strain, none of them exceeding their perception threshold.

In terms of compounds that can negatively affect the sensory characteristics of the beer (acetaldehyde, DMS, acetone, and VDKs), a great reduction of these compounds was observed after 48 h of aeration. Acetaldehyde and VDKs are compounds that are found in higher concentrations in green beer, giving unpleasant “young” or “green” off-tastes, as well as participating with phenolics in the formation of beer haze [58]. Therefore, as the beer samples were not matured, the diacetyl concentrations obtained, despite having decreased significantly during fermentation with aeration, continued to be above their threshold (0.15 mg L^−1^) [49]. At first sight, a reduction in diacetyl concentration is contradictory since an increase in cell growth would imply an increase in the production of valine, the by-product of which during fermentation is diacetyl. However, these results were also obtained by Brányik et al., during their tests under different conditions [55]. After maturation, diacetyl is reduced by yeast to acetoin and 2,3-butanediol, with relatively high thresholds (50 mg L^−1^ and 4500 mg L^−1^, respectively) [49]. Strains CLI 101 and CLI 1057 showed a high significance in diacetyl and 2,3-pentanedione production, respectively, which were significantly reduced in the aerated fermentation. DMS can also produce undesirable aroma impressions, the concentrations of which initially depend on the malt and hops used but can be increased by yeast metabolism from dimethyl sulfoxide (DMSO) [59,60].

For experiment C, sequential fermentation, the production of volatile compounds, such as esters and higher alcohols, was increased compared with pure culture. Still, only ethyl acetate, isoamyl acetate, isoamyl alcohols, ethyl caproate, and 4VG exceeded the sensory threshold. All three strains showed a higher significance compared to the A and B experiments. In addition, strain CLI 512 (*H. guilliermondii*) produced higher concentrations of ethyl acetate than the control strain S-04. *H. guilliermondii* species, as well as *T. delbrueckii* species, have previously been described as suitable for sequential or mixed fermentation due to the fruity character they can bring to beer, enhancing their aromatic profile [7,13,61]. However, the species *Z. bailii* has not been extensively studied in beer, its references being related to the production of beer with low ethanol content or in sequential culture with *Saccharomyces* to ferment maltose and thus avoid the wort-like off-flavors created by residual wort sugars [26,62]. The concentration of higher alcohols decreases concerning the pure culture of *Saccharomyces* S-04 strain, the production of amyl alcohols being remarkable since they exceed the threshold value (70 mg L^−1^). The values obtained ranged from 70.23 to 110.01 mg L^−1^, while for strain S-04, they were 120.47 mg L^−1^. The genus *Hanseniaspora*, although described as a producer of low concentrations of higher alcohols in pure culture [13], can be observed in sequential culture as the one that reduced, to a lesser extent, the higher alcohols produced by S-04. On the other hand, none of the strains studied sequentially produced concentrations above the perception threshold for the other higher alcohols. Apart from higher alcohols, *H. guilliermondii* (CLI 512) had the highest acetate ester content, even in pure culture, and mostly attributed to higher levels of ethyl acetate (96.37 mg L^−1^), being indeed significant values and higher than those of the control strain S-04. These data suggest a lower performance for ethyl acetate production or higher esterase activity linked to the yeast strain. These species have also been identified as major producers of ethyl acetate in wine [45,63]. It should also be noted that high concentrations of this ester could produce solvent-like aromas instead of fruity aromas [64]. Thus, it would be necessary to conduct a sensory analysis of the beer to determine whether these would be detected. The values for isoamyl acetate were higher than the threshold value of 1.2 mg L^−1^, with 5.87 mg L^−1^ for *H. guilliermondii* (CLI 512), 5.01 mg L^−1^ for *Z. bailee* and 4.27 mg L^−1^ for *T. delbrueckii*, thus showing significance for all three strains. These concentrations were considerably higher than those from the pure culture, where levels were below detection limits. This is due to the production of isoamyl acetate by the *Saccharomyces* S-04 strain, whose concentrations in pure culture were 9.55 mg L^−1^ and therefore reduced in sequential culture. Other compounds with noticeable differences from the pure culture were DMS and VDKs. DMS concentration increased above that obtained in pure *Saccharomyces* and non-*Saccharomyces* cultures for strains CLI 512 and CLI 622, with strain CLI 622 being significantly different from the other strains. However, for the sequentially fermented beer with strain CLI 895, these levels were similar to the control strain S-04. These concentrations were even higher than those obtained in pure culture and fermentation with aeration. Therefore, the maturation of green beer would be necessary for the adjustment of these flavors. The same would be true for VDKs, where the values obtained are strain-dependent, and maturation would be necessary to reduce them below the threshold.

For all three experiments, 4VG production remained similar. However, the concentrations obtained by strain CLI 1057 in both pure culture and aerated fermentation produced considerably high levels (1.65 and 1.86 mg L^−1^, respectively) above the flavor threshold (0.3 mg L^−1^) [29], even higher than those of commercial beers (0.13 to 1.11 mg L^−1^) [65]. This strain showed a high significance in both experiments. Wild *Saccharomyces* yeast isolates tend to produce high concentrations of phenolic compounds, giving the beer a clove-like aroma [66]. This revealed that the *S. cerevisiae* strain CLI 1057 may be a candidate for the production of beers with a phenolic profile.

Therefore, within the trials performed under different fermentation conditions, different species and/or strains would stand out in the production of aromatic compounds. In experiment A (pure culture), the yeast strain CLI 1057 (*S. cerevisiae*) stands out for its high production of esters, such as isoamyl acetate, amyl alcohols, and 4VG, above its thresholds. Regarding experiment B (fermentation with aeration), for the production of esters (ethyl acetate), strain CLI 512 is outstanding, and in terms of amyl alcohols, strains CLI 895 and CLI 1057 stand out. In this case, strain CLI 1057 also remains remarkable in terms of 4VG production. Finally, in experiment C (sequential fermentation), there was a notable increase in the production of esters and higher alcohols for the three study strains, with strain CLI 512 producing the highest concentrations compared to the control strain S-04.

### 3.4. Principal Components Analysis (PCA)

**Figure 3:**
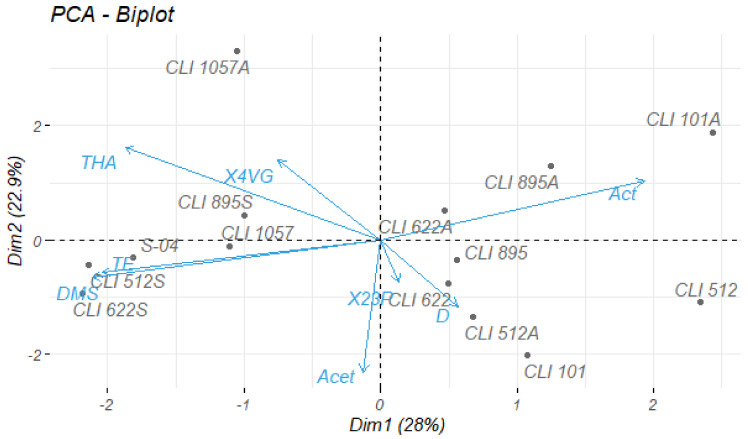
Projection of the beers on the axes formed by the principal components 1 and 2. Each object is the average of the two corresponding experimental beers. Act, acetaldehyde; DMS, dimethyl sulfide; Acet, acetone; D, diacetyl; X23P, 2,3-pentanedione; THA, total higher alcohols, TE, total esters; X4VG, 4-vinyl guaiacol. Yeast strains followed by A are related to fermentation with aeration, followed by S are related to sequential fermentation, and with no letter are related to pure cultures.

In order to visualize the differences in volatile compounds depending on the experiment and yeast strain, a Principal Components Analysis (PCA) was performed using the mean of the repetitions (Figure 3).

The final data matrix was composed of fourteen objects and eight variables: acetaldehyde, DMS, acetone, diacetyl, 2,3-pentanedione, total higher alcohols (sum of methanol, propanol, isobutanol, and amyl alcohols), total esters (sum of ethyl formate, ethyl acetate, ethyl propionate, isoamyl acetate, and ethyl caproate), and 4VG. The two main components accounted for 50.9% of the variability of the original data.

In general, a clear clustering has been observed for most of the strains studied depending on the experiment carried out. Yeast strains used for the fermentation in pure culture are situated on the negative side of the PC2, with most of them grouped on the positive side of PC1, which is related to the high production of diacetyl and 2,3-pentanedione. Beers obtained in fermentation with aeration showed a high trend in terms of acetaldehyde production, which is why they were located in the positive zones of PC1 and PC2. Strain CLI 1057 differs from the rest, both in pure culture and with aeration, due to its high 4VG production. Finally, the strains CLI 512, CLI 622, and CLI 895 used in the sequential culture fermentation were shown on the negative side of PC1, close to the control strain S-04, and associated with the production of DMS and total esters.

## 4. Conclusions

This paper presents the fermentative behavior of non-conventional wine yeast under three specific conditions. The non-conventional strains evaluated could not metabolize maltose, maltotriose, and sucrose for some strains (CLI 101, CLI 512, CLI 622, CLI 691, and CLI 901). However, yeast strain CLI 1057 (*S. cerevisiae*) started fermenting maltose with aeration due to the Kluyver effect presented by this strain for maltose sugar. It is clear from the present work that fermentation with aeration not only produced variations in the fermentation of available sugars in the wort but also in the final concentration of higher alcohols and esters, with a strain-dependent relationship. These differences are especially interesting because fermentations with the different non-conventional strains under pure o aerated culture produce low-alcohol beers (1.07 to 1.26% *v*/*v*) with an important effect on the flavor of fermented products. Furthermore, for strain CLI 1057, the alcohol content could be increased with aeration to obtain a less sweet beer as well as a fruitier and phenolic profile. Therefore, the control of aeration during fermentation is a critical point not only in guiding yeast metabolism but also in the production of volatile compounds that contribute to the organoleptic character of the beer. For that reason, strain CLI 1057 could be used for producing both low-ethanol beer and beer with higher alcohol content and phenolic characteristics.

Non-*Saccharomyces* yeasts are characterized by producing higher amounts of higher alcohols and esters. Therefore, their use to enhance the flavor of the beer is receiving significant research attention. The results obtained demonstrate that some non-*Saccharomyces* strains can increase the flavor complexity of sequentially fermented beers (CLI 512), but further studies should be conducted to improve brewing processes.

The perception of aroma compounds depends on multiple factors, known as the “matrix effect”, as well as synergisms or antagonisms. For this reason, it is necessary to study the sensory profile of the beers brewed in order to evaluate the positive or negative effect of the high concentrations of some compounds, such as ethyl acetate, produced in the different experiments.

## Figures and Tables

**Table 1:** Residual sugars (g/100 mL) and ethanol content in beers obtained after fermentation with six non-conventional yeast and the control S-04 strain in trials with 400 mL of wort.

Yeast Strains	Glucose	Fructose	Sucrose	Maltose	Maltotriose	Ethanol % (*v*/*v*)	Final Apparent Extract % (m/m)	Apparent Attenuation (%)
CLI 101	0.01 ± 0.00 ^b^	0.01 ± 0.00 ^b^	0.33 ± 0.00 ^b^	6.56 ± 0.08 ^b^	2.02 ± 0.01 ^a^	1.16 ± 0.33 ^bc^	13.29 ± 0.28 ^cd^	20.59
CLI 622	0.01 ± 0.00 ^b^	0.02 ± 0.00 ^b^	0.38 ± 0.01 ^a^	7.05 ± 0.02 ^a^	1.99 ± 0.01 ^ab^	1.07 ± 0.19 ^bc^	13.98 ± 0.12 ^b^	16.18
CLI 691	0.01 ± 0.00 ^b^	0.02 ± 0.00 ^b^	0.38 ± 0.01 ^a^	7.00 ± 0.04 ^a^	1.98 ± 0.03 ^ab^	1.07 ± 0.19 ^bc^	13.91 ± 0.19 ^bc^	16.18
CLI 895	0.01 ± 0.00 ^b^	0.02 ± 0.00 ^b^	0.01 ± 0.00 ^c^	7.07 ± 0.04 ^a^	2.05 ± 0.04 ^a^	1.24 ± 0.44 ^b^	13.11 ± 0.09 ^d^	22.06
CLI 901	1.61 ± 0.05 ^a^	0.67 ± 0.01 ^a^	0.38 ± 0.01 ^a^	6.93 ± 0.04 ^a^	1.95 ± 0.01 ^b^	0.01 ± 0.00 ^c^	15.98 ± 0.21 ^a^	2.94
CLI 1057	0.01 ± 0.00 ^b^	0.02 ± 0.00 ^b^	0.01 ± 0.00 ^c^	6.96 ± 0.13 ^a^	1.99 ± 0.01 ^ab^	1.26 ± 0.47 ^b^	13.12 ± 0.08 ^d^	22.06
S-04	0.01 ± 0.00 ^b^	0.02 ± 0.00 ^b^	0.01 ± 0.00 ^c^	0.02 ± 0.00 ^c^	0.04 ± 0.00 ^c^	5.69 ± 0.23 ^a^	1.95 ± 0.01 ^e^	88.24

Data are means ± standard deviations of two independent samples. Data with different superscript letters within each column are significantly different (Tukey tests: *p* < 0.05).

**Table 2:** The main analytical characteristics of six non-conventional strains and S-04 in 400 mL fermentation analyzed with GC.

Yeast Strains	Acetaldehyde (mg L^−1^)	DMS (μg L^−1^)	Acetone (mg L^−1^)	Total Higher Alcohols (mg L^−1^)	Total Esters (mg L^−1^)
CLI 101	13.05 ± 4.17 ^b^	<17.00 ^b^	1.48 ± 0.02	156.55 ± 13.51 ^b^	7.43 ± 3.05 ^b^
CLI 622	2.70 ± 0.28 ^b^	<17.00 ^b^	1.12 ± 0.10	121.45 ± 5.30 ^bc^	2.17 ± 0.14 ^b^
CLI 691	2.40 ± 0.14 ^b^	33.80 ± 10.61 ^a^	1.04 ± 0.09	143.40 ± 2.97 ^bc^	3.12 ± 0.21 ^b^
CLI 895	24.45 ± 4.88 ^b^	<17.00 ^b^	1.13 ± 0.29	143.55 ± 14.07 ^bc^	7.95 ± 1.70 ^b^
CLI 901	0.45 ± 0.07 ^b^	17.00 ± 0.01 ^b^	1.02 ± 0.35	5.00 ± 0.00 ^d^	0.55 ± 0.00 ^b^
CLI 1057	>30 ^a^	17.35 ± 0.49 ^b^	0.96 ± 0.06	109.00 ± 11.59 ^c^	3.99 ± 0.32 ^b^
S-04	>30 ^a^	<17.00 ^b^	0.97 ± 0.08	304.05 ± 15.34 ^a^	80.37 ± 17.65 ^a^

Data are means ± standard deviations of two beer samples. Data with different superscript letters within each column are significantly different (Tukey tests: *p* < 0.05).

**Table 3:** The main volatile compounds in the beer are produced by the different yeast strains (mg L^−1^) under different experimental conditions (A, B, C).

Compounds	CLI 101	CLI 512	CLI 622	CLI 895	CLI 1057	S-04	Experiment
Acetaldehyde	0.64 ± 0.21	4.04 ± 4.76	8.45 ± 10.94	1.55 ± 0.10	0.71 ± 0.06	0.93 ± 0.04	A
	4.19 ± 1.90	1.26 ± 0.34	1.59 ± 0.33	2.55 ± 0.15	1.78 ± 0.89		B
		1.11 ± 0.04	0.89± 0.05	0.95 ± 0.30			C
DMS	**13.48 ± 2.87 ^efg^**	**1.94 ± 0.47 ^gh^**	**19.07 ± 3.51 ^def^**	**27.26 ± 4.02 ^cd^**	**50.84 ± 1.70 ^ab^**	**25.11 ± 2.97 ^de^**	A
(μg L^−1^)	nd	nd	**26.12 ± 5.28 ^cde^**	**2.16 ± 0.77 ^cd^**	**9.54 ± 1.99 ^fgh^**		B
		**38.35 ± 0.53 ^bc^**	**62.66 ± 8.43 ^a^**	**28.14 ± 0.69 ^gh^**			C
Acetone	0.30 ± 0.23	0.39 ± 0.06	0.28 ± 0.03	0.23 ± 0.00	0.22 ± 0.25	0.30 ± 0.10	A
	0.06 ± 0.01	0.29 ± 0.09	0.15 ± 0.04	0.14 ± 0.05	0.10 ± 0.02		B
		0.26 ± 0.04	0.27 ± 0.03	0.12 ± 0.09			C
Diacetyl	**478.89 ± 146.30 ^a^**	**56.43 ±0.71 ^b^**	**21.35 ± 1.14 ^b^**	**57.16 ± 2.53 ^b^**	**96.46 ± 53.63 ^b^**	**5.57 ± 2.53 ^b^**	A
	**29.25 ± 14.89 ^b^**	**49.88 ± 2.20 ^b^**	**17.80 ± 10.50 ^b^**	**22.40 ± 9.28 ^b^**	**37.81 ± 12.57 ^b^**		B
		**35.74 ± 18.32 ^b^**	**18.03 ± 4.84 ^b^**	**55.69 ± 5.80 ^b^**			C
2,3-	**4.96 ± 0.59 ^c^**	**28.30 ± 6.40 ^abc^**	**4.41 ± 0.26 ^c^**	**5.52 ± 2.64 ^bc^**	**40.65 ± 24.37 ^a^**	0.69 ± 0.12 ^c^	A
pentanedione	**3.47 ± 2.98 ^c^**	**37.52 ± 13.81 ^ab^**	**2.24 ± 1.18 ^c^**	**1.60 ± 0.42 ^c^**	**7.71 ± 3.20 ^bc^**		B
		**8.08 ± 5.34 ^bc^**	**5.03 ± 1.13 ^c^**	**17.74 ± 4.51 ^ab^**			C
Ethyl formate	0.01 ± 0.00 ^d^	0.01 ± 0.02 ^d^	nd	0.02 ± 0.01 ^cd^	0.04 ± 0.01 ^cd^	0.09 ±0.01 ^ab^	A
	0.01 ± 0.01 ^d^	nd	0.01 ± 0.00 ^b^	0.01 ± 0.00 ^d^	0.10 ± 0.03 ^a^		B
		0.06 ± 0.01 ^abc^	0.06 ± 0.00 ^bc^	0.06 ± 0.00 ^bc^			C
Ethyl acetate	1.93 ± 0.66 ^d^	10.70 ± 3.03 ^d^	2.51 ± 0.07 ^d^	4.79 ± 0.17 ^d^	5.69 ± 0.22 ^d^	**78.97 ± 10.57 ^ab^**	A
	3.86 ± 0.12 ^d^	**50.58 ± 7.55 ^c^**	1.28 ± 0.20 ^d^	6.06 ± 1.90 ^d^	15.23 ± 4.04 ^d^		B
		**96.37 ± 10.47 ^a^**	**71.33 ± 6.89 ^b^**	**50.21 ± 0.51 ^c^**			C
Methanol	3.31 ± 0.24 ^abcd^	1.90 ± 0.36 ^cd^	2.62 ± 0.03 ^bcd^	2.85 ± 0.11 ^bcd^	3.12 ± 0.49 ^bcd^	4.87 ± 0.05 ^a^	A
	1.85 ± 0.26 ^d^	1.76 ± 0.15 ^d^	2.43 ± 0.65 ^bcd^	2.55 ± 0.98 ^bcd^	2.70 ± 0.14 ^bcd^		B
		3.70 ± 0.13 ^ab^	3.51 ± 0.48 ^abc^	2.92 ± 0.36 ^bcd^			C
Ethyl	0.00 ± 0.00 ^d^	0.14 ± 0.12 ^cd^	0.02 ± 0.00 ^e^	0.13 ± 0.01 ^cd^	0.01 ± 0.01 ^d^	1.18 ± 0.07 ^a^	A
propionate	0.02 ± 0.01 ^d^	0.08 ± 0.08 ^cd^	0.02 ± 0.01 ^d^	0.08 ± 0.00 ^cd^	0.17 ± 0.01 ^cd^		B
		0.26 ± 0.10 ^c^	0.21 ± 0.01 ^cd^	0.54 ± 0.08 ^b^			C
Propanol	2.04 ± 0.20 ^d^	5.73 ± 1.83 ^cd^	6.45 ± 2.55 ^cd^	7.45 ± 0.15 ^cd^	13.14 ± 0.66 ^c^	44.36 ± 2.37 ^ab^	A
	6.18 ± 0.66 ^cd^	5.28 ± 3.61 ^cd^	10.07 ± 1.90 ^cd^	11.56 ± 1.66 ^cd^	40.63 ± 2.38 ^ab^		B
		50.15 ± 6.42 ^a^	37.96 ± 3.45 ^b^	49.82 ± 2.28 ^a^			C
Isobutanol	20.15 ± 1.42 ^ef^	8.43 ± 2.09 ^hi^	8.57 ± 2.34 ^ghi^	4.48 ± 0.37 ^i^	18.91 ± 1.67 ^efgh^	52.73 ± 1.44 ^b^	A
	19.41 ± 1.65 ^efg^	11.18 ± 4.03 ^fghi^	21.80 ± 4.67 ^ef^	23.54 ± 3.91 ^e^	93.43 ± 0.34 ^a^		B
		44.57 ± 3.01 ^bc^	24.50 ± 2.74 ^de^	35.39 ± 3.96 ^cd^			C
Isoamyl	0.01 ± 0.00 ^e^	0.92 ± 0.02 ^e^	0.01 ± 0.01 ^e^	0.01 ± 0.00 ^e^	**2.46 ± 0.14 ^cd^**	**9.55 ± 1.23 ^a^**	A
acetate	0.01 ± 0.00 ^e^	0.15 ± 0.21 ^e^	0.14 ± 0.04 ^e^	0.03 ± 0.01 ^e^	0.92 ± 0.09 ^de^		B
		**5.87 ± 1.45 ^b^**	**5.01 ± 0.66 ^b^**	**4.27 ± 0.06 ^bc^**			C
Amyl alcohols	29.71 ± 1.16 ^ef^	16.63 ± 4.29 ^f^	16.53 ± 4.71 ^f^	39.40 ± 2.43 ^e^	**70.19 ± 2.40 ^d^**	**120.47 ± 1.79 ^b^**	A
	31.85 ± 1.98 ^ef^	31.51 ± 1.40 ^ef^	48.92 ± 6.92 ^e^	**89.55 ± 11.59 ^cd^**	**201.41 ± 3.14 ^a^**		B
		**110.01 ± 0.93 ^c^**	**70.23 ± 5.46 ^d^**	**90.20 ± 7.70 ^cd^**			C
Ethyl caproate	0.00 ± 0.00 ^e^	0.01 ± 0.01 ^e^	0.00 ± 0.00 ^e^	0.01 ± 0.00 ^e^	0.12 ± 0.03 ^cd^	**0.32 ± 0.03 ^a^**	A
	nd	0.00 ± 0.00 ^e^	0.00 ± 0.00 ^e^	nd	0.06 ± 0.00 ^de^		B
		**0.22 ± 0.02 ^b^**	**0.17 ± 0.01 ^bc^**	**0.23 ± 0.04 ^b^**			C
4VG	0.23 ± 0.02 ^b^	0.16 ± 0.02 ^b^	0.20 ± 0.04 ^b^	0.20 ± 0.03 ^b^	**1.65 ± 0.95 ^a^**	0.18 ± 0.02 ^b^	A
	0.18 ± 0.01 ^b^	0.16 ± 0.02 ^b^	0.20 ± 0.05 ^b^	0.21 ± 0.05 ^b^	**1.86 ± 0.00 ^a^**		B
		0.16 ± 0.02 ^b^	0.16 ± 0.01 ^b^	0.15 ± 0.00 ^b^			C

Data with different superscript letters within each row are significantly different (Tukey tests: *p* < 0.05). Compounds above their threshold levels are marked in bold. Data are means ± standard deviations of two independent samples. Experiment A, pure culture; Experiment B, fermentation with aeration; Experiment C, sequential fermentation.

## Data Availability

Data is contained within the article.
